# Therapeutic Potential of ω-3 Polyunsaturated Fatty Acids in Human Autoimmune Diseases

**DOI:** 10.3389/fimmu.2019.02241

**Published:** 2019-09-27

**Authors:** Xiaoxi Li, Xinyun Bi, Shuai Wang, Zongmeng Zhang, Fanghong Li, Allan Z. Zhao

**Affiliations:** ^1^The School of Biomedical and Pharmaceutical Sciences, Guangdong University of Technology, Guangzhou, China; ^2^Department of Immunology, Nanjing Medical University, Nanjing, China

**Keywords:** ω-3 polyunsaturated fatty acids, autoimmune diseases, inflammation, eicosanoids, mTOR-the mammalian target of rapamycin

## Abstract

The recognition of ω-3 polyunsaturated acids (PUFAs) as essential fatty acids to normal growth and health was realized more than 80 years ago. However, the awareness of the long-term nutritional intake of ω-3 PUFAs in lowering the risk of a variety of chronic human diseases has grown exponentially only since the 1980s (1, 2). Despite the overwhelming epidemiological evidence, many attempts of using fish-oil supplementation to intervene human diseases have generated conflicting and often ambiguous outcomes; null or weak supporting conclusions were sometimes derived in the subsequent META analysis. Different dosages, as well as the sources of fish-oil, may have contributed to the conflicting outcomes of intervention carried out at different clinics. However, over the past decade, mounting evidence generated from genetic mouse models and clinical studies has shed new light on the functions and the underlying mechanisms of ω-3 PUFAs and their metabolites in the prevention and treatment of rheumatoid arthritis, systemic lupus erythematosus (SLE), multiple sclerosis, and type 1 diabetes. In this review, we have summarized the current understanding of the effects as well as the underlying mechanisms of ω-3 PUFAs on autoimmune diseases.

## Introduction

Polyunsaturated fatty acids (PUFAs) can be divided into two major classes: ω-3 PUFAs and ω-6 PUFAs, with the primary structural difference at the positions of their double bonds on the carbon chain. ω-6 PUFAs have the first double bonds starting at the sixth carbon, while ω-3 PUFAs starting at the third carbon, from the methyl end of the carbon chain (the ω-carbon) (3). The two major ω-6 PUFAs that are typically consumed in the diet are linoleic acid (18:2; ω-6; LA) and arachidonic acid (20:4; ω-6; AA). Western diets are dominated by ω-6 PUFAs but contain only small amounts of ω-3 PUFAs with the ratio of ω-6/ω-3 reaching as high as 20–30 (4). The three major ω-3 PUFAs are α-linolenic acid (18:3; ω-3; α-LA), eicosapentaenoic acid (20:5; ω-3; EPA), and docosahexaenoic acid (22:6; ω-3; DHA). The α-LA (18:3; ω-3) should not be confused with γ-linolenic acid (GLA) that is also 18:3 but an ω-6 fatty acid. LA (precursor to the ω-6 series of fatty acids) and α-LA (precursor to the ω-3 series of fatty acids) are considered essential fatty acids because they cannot be synthesized in mammals. Starting with LA, mammals can sequentially synthesize GLA via Δ6-desaturase (Fads2) then AA through elongase and Δ5-desaturase (Fads1) (5–7). On the other hand, α-LA is converted to stearidonic acid (C18:4; ω-3) by Δ6-desaturase; stearidonic acid is then elongated and converted to EPA via Δ5-desaturase. EPA add two carbons via elongase to form docosapentaenoic acid (22:5; ω-3; DPA), then add two more carbons via elongase followed by desaturation by Δ6-desaturase to produce tetracosahexaenoic acid (24:6; ω-3); the subsequent, removal of two carbons by β-oxidation yields DHA. Although Δ6-desaturase is widely considered as rate-limiting for the sequential synthesis from ALA to DHA (8–11), a recent study also revealed that elongase reactions (primarily mediated by elongase-5 in humans) were control points as well (12). These enzymatic constraints limit the tissue levels of EPA and DHA. Consequently, we still need to rely on dietary intake to meet the physiological demands of EPA and DHA.

The beneficial effects of ω-3 PUFAs are mediated by themselves as well as by their bioactive metabolites, such as resolvins, protectins, and maresins. Among them, the most studied anti-inflammation metabolites are resolvins which have two classes. E-series resolvins include RvE1, RvE2, and RvE3, all of which are synthesized from EPA by such enzymes as cytochrome P450 (CYP 450), cyclocygenase-2 (COX-2), and 15-lipoxygenase (15-LOX) (13, 14). The D series resolvins (RvD1–RvD6) are derived from DHA by such enzymes as COX-2, 12/15 LOX, and 5-LOX (13, 15–17). These metabolites from ω-3 PUFAs compete with those of ω-6 PUFAs to promote the resolution of the inflammatory cycle (18, 19), and have been increasingly recognized as the important players in the attenuation of inflammation and regulation of autoimmunity (18, 19).

Mounting evidence from both human and animal studies has demonstrated that ω-3 PUFAs, primarily EPA and DHA, can suppress inflammation and have beneficial roles in a variety of human diseases, including autoimmune diseases, diabetes, cancers, Alzheimer's disease (AD), and stroke (3, 20–26). Fish oil is the oil derived from the tissues of oily ocean fish. The main ω-3 PUFAs in fish oil are EPA and DHA. Researchers often use fish oil as the source of ω-3 PUFAs in clinical interventions. Although the effects of ω-3 PUFAs on these diseases have been extensively investigated in the past few decades, a series of recent studies involving the usage of a transgenic genetic model, fat-1 (or mfat-1) transgenic mice, have shed new light into the mechanisms underlying the protective effects of ω-3 PUFAs. The fat-1 transgenic mice, initially generated in Kang's lab (27) [and later the mfat-1 mouse model independently designed by our group (28)] carry a globally expressed *Caenorhabditis elegans* fat-1 transgene that encodes an ω-3 fatty acid desaturase. The FAT-1 enzyme can convert ω-6 PUFAs to the corresponding ω-3 forms by adding a double bond to the ω-3 position, thus allowing endogenous production of ω-3 PUFAs while reducing the ω-6: ω-3 ratio in tissues without special dietary adjustment (27, 28). The fat-1 transgenic model allows researchers to bypass the traditional lengthy dietary approach of feeding fish-oil to the animals, and have been widely applied to the studies related to autoimmune diseases, tumorigenesis, metabolic, and cardiovascular diseases, as well as neurological diseases (24, 27, 29, 30).

In this review, we choose to focus on the current understanding of the effects of ω-3 fatty acids on such debilitating diseases as rheumatoid arthritis (RA) (31), systemic lupus erythematosus (SLE) (32), type 1 diabetes (T1D) (33), and multiple sclerosis (MS) (34). Although there have been a large body of clinical research articles related to the application of ω-3 PUFAs in different indications, perhaps it should come with no surprise that not all published studies reached the same conclusions. The reasons behind such discrepancy are still unclear although different dose, source, and duration of ω-3 PUFAs treatment may have all played roles in the clinical outcomes. Unfortunately, many of these studies did not report the blood concentrations of EPA/DHA of the enrolled patients, making it difficult to evaluate if the enrolled patients for their studies have indeed gained sufficient EPA/DHA. Thus, for the benefit of readers, we have offered several formatted tables (by no means complete, but certainly representative) detailing the number of patients, dose, duration, and outcomes of each trial (Tables 1–4).

**Table 1:** Interventional studies with ω-3PUFAs in patients with rheumatoid arthritis (RA).

**Study**	***n***	**Study design**	**Results**
Kremer et al. (35)	37 patients with RA	17 patients took an experimental diet with a daily supplement of 1.8 g EPA for 12 weeks. 20 patients took a control diet with a lower polyunsaturated to saturated fat ratio and a placebo supplement for 12 weeks.	The experimental group had deteriorated significantly in patient and physician global evaluation of disease activity, pain assessment, and number of tender joints at 12th week and the following 1–2 months after stopping the diets.
Kremer et al. (36)	49 patients with active RA	20 patients consumed daily dietary supplements of 27 mg/kg EPA and 18 mg/kg DHA (low dose), 17 patients ingested 54 mg/kg EPA and 36 mg/kg DHA (high dose), and 12 patients ingested olive oil capsules containing 6.8 mg of oleic acid.	Significant decreases in the number of swollen joints were noted in the low and high-dose groups at weeks 12, 18, 24. Clinical benefits of dietary supplementation with ω-3 fatty acids are more commonly observed in patients consuming higher dosages of fish oil and olive oil is also associated with certain changes in immune function.
Veselinovic et al. (37)	60 patients with active RA	60 patients with active RA were involved in a prospective, randomized trial of a 12-week supplementation with fish oil (group I), fish oil with primrose evening oil (group II), or with no supplementation (group III). Clinical and laboratory evaluations were done at the beginning and at the end of the study.	The Disease Activity Score 28 (DAS 28 score), number of tender joints and visual analog scale (VAS) score decreased notably after supplementation in groups I and II (*p* < 0.001). In plasma phospholipids the n-6/n-3 fatty acids ratio declined from 15.47 ± 5.51 to 10.62 ± 5.07 (*p* = 0.005), and from 18.15 ± 5.04 to 13.50 ± 4.81 (*p* = 0.005) in groups I and II, respectively.
Beyer et al. (38)	78 RA patients (age 57 ± 12 y, disease duration 15 ± 11 y)	RA outpatients (age ≥35 y) were consecutively recruited. Rheumatologic clinical data were collected and periodontal status obtained. A food frequency questionnaire (FFQ) was used to estimate fish and supplement intake.	Seafood intake in accordance with nutritional recommendations was associated with better RA disease outcome (*P* = 0.008). An ω-3 index >8, present in 14% of the patients, correlated with a more desirable patient global health assessment scored on a visual analog scale (VAS; *P* = 0.004).
Van der Tempel et al. (39)	16 patients with RA	Patients were randomly allocated to receive each day either 12 capsules of fractionated fish oil (2.04 g DPA, 1.32 g DHA) or fractionated coconut oil flavored with fish aroma as placebo for 12 weeks.	Dietary fish oil supplementation is effective in suppressing joint swelling index and duration of early morning stiffness. Other clinical indices improved but did not reach statistical significance. The mean neutrophil leucotriene B4 production *in vitro* showed a reduction after 12 weeks of fish oil supplementation. Leucotriene B5 production rose to substantial quantities during fish oil treatment.
Lee et al. (40)	183 RA patients and 187 placebo-treated RA controls were included in this meta-analysis	The authors surveyed RCTs that examined the effects of ω-3 PUFAs on clinical outcomes in RA patients using MCCTR and by performing manual searches. Meta-analysis of RCTs was performed using fixed and random effects models.	The use ofω-3 PUFAs at dosages >2.7 g/day for >3 months reduces NSAID consumption by RA patients.
Espersen et al. (41)	32 patients with active RA	A 12-week double-blind, randomized study of dietary supplementation with ω-3 fatty acids (3.6 g per day) or placebo.	Dietary supplementation with ω-3 fatty acids results in significantly reduced plasma IL-1 beta levels in patients with rheumatoid arthritis, and the clinical status of the patients was improved in fish oil group.

*NSAID, Non-steroidal anti-inflammatory drug; RA, Rheumatoid arthritis; DPA, Docosapentaenoic acid; RCTs, Randomized controlled trials; MCCTR, Medline and the cochrane controlled trials register; NSAID, Non-steroidal anti-inflammatory drugs; FFQ, Food-frequency questionnaire; DMARD, Disease-modifying anti-rheumatic drug; ACR, American college of rheumatology*.

## ω-3 PUFAs and Rheumatoid Arthritis

Rheumatoid arthritis (RA) is a chronic autoimmune disease manifested by swollen and painful joints, bone erosion, and functional impairment. The joint lesions are characterized by infiltration of T lymphocytes, macrophages, and B lymphocytes into the synovium, as well as by synovial inflammation induced by PUFAs metabolites, cytokines, and matrix metalloproteinases (58–60). The intervention of RA with ω-3 PUFAs has attracted increasing attention. One of the earliest studies about ω-3 PUFAs' impact on rheumatoid arthritis was reported in Lancet in 1985 (35) (Table 1). In this pilot study, seventeen RA patients consumed a daily supplement of 1.8 g of EPA for 12 weeks. At the end of treatment, the EPA intervention significantly shortened morning stiffness time than the control group at 12 weeks and lessened the number of tender joints at 12 weeks from baseline. Several years following this study, Kremer et al. reported in 1990 that the number of tender joints significantly improved from baseline after both the low- and high-dose intervention with fish oil supplement (36) (Table 1). In addition to such beneficial effects, an ω-6 PUFAs-derived metabolite, leukotriene B4 (LTB_4_), generated by the neutrophils of the fish oil-treated patients, decreased significantly (36) (Table 1). In a prospective study, sixty patients with active RA were enrolled in a randomized 12-week trial, who were given either fish oil supplement, or fish oil supplement with primrose evening oil (enriched in GLA), or no intervention (37) (Table 1). Daily supplementation of fish oil alone or in combination with primrose evening oil significantly improved the number of tender joints and visual analog scale (VAS) score. There was a sharp decline in the ratio of ω-6/ω-3 fatty acids in the plasma samples from the groups of subjects receiving fish oil, which was strongly linked to the clinical improvements. Beyer et al. reported in a nutrition study involving 78 RA patients, of which 58% had active RA, that consuming the recommended seafood intake was correlated with better RA disease outcome (38) (Table 1). In a separate study, sixteen patients received a daily dose of 2.04 g EPA and 1.32 g DHA for 12 weeks, the results also showed a significant reduction in joint swelling index and duration of early morning stiffness as well as a decrease of LTB4 production by the neutrophils isolated from patients' blood (39) (Table 1). A meta-analysis of 23 similar clinical studies revealed a fairly consistent finding that ω-3 PUFAs had a beneficial effect on joint swelling, pain and morning stiffness, and that there was a significant reduction in the required dose of steroidal anti-inflammatory drugs (40) (Table 1).

In an animal model study, fish-oil feeding in mice delayed the onset and reduced the incidence and severity of type II collagen-induced arthritis compared with the vegetable oil-fed group (61). In the susceptible DBA/1 mouse strain, daily intake of marine ω-3 PUFAs in the form of phospholipids delayed the onset of arthritis, decreased the severity, reduced paw swelling, and knee joint pathology in collagen-induced arthritis (62). Both EPA and DHA have also been shown to suppress *Streptococcal*-induced arthritis in rats, although EPA appeared to be more effective than DHA (63). As additional proof, endogenous production of ω-3 PUFAs in the fat-1 transgenic mice drastically attenuated arthritis as well as local and systemic levels of inflammatory cytokines following the establishment of RA, whereas the wild type control mice developed overt arthritis (64). Thus, in both animal models and patients, ω-3 PUFAs were able to decrease not only the incidence but also the severity of RA.

The mechanisms underlying ω-3 PUFAs-initiated regulation of immunity in RA can be several. Most studies focused on molecular mediators, including cytokines, metabolites, and reactive oxygen species; others have interrogated the roles of immune cells such as T cells and antigen presenting cells. In the study by Espersen et al. (41) (Table 1), the level of interleukin-1β (IL-1β) in plasma was significantly suppressed in the patients who consumed fish oil by week 6 of the trial, and a further significant decrease was observed a few weeks later. Even in healthy volunteers, a clinical study involving supplementation of fish oil has demonstrated reduction in tumor necrosis factor-alpha (TNF-α), IL-1β, and interleukin-6 (IL-6) in endotoxin-stimulated monocytes cells (65), although the findings from a few other studies did not corroborate these results (66–68). Recently, interleukin-17 (IL-17) has been proposed to be a key cytokine in the onset and development of RA (69, 70). Studies using fish oil supplement found that DHA and EPA have anti-inflammation benefit by reducing the population of interferon-γ (IFN-γ) and IL-17-producing CD4^+^ T cells in humans and animals (71–74). Thus, suppression of inflammatory cytokines is likely an important contributing factor to the amelioration of clinical signs and symptoms in RA patients. In cultured cells, studies also reported that EPA and DHA could inhibit the production of TNF-α, IL-1β, and IL-6, the classic panel of pro-inflammatory cytokines (75–78). The results derived from these clinical and cellular studies were also corroborated by similar findings in other animal models (24, 30). For example, the fat-1 transgenic mice showed strong anti-inflammatory effects that rendered tissues strongly resistant to cytokines (IL-1β and TNF-α)-induced cell death (24, 30). The inflammatory metabolites, prostaglandin E2 (PGE_2_) and LTB_4_, were sharply decreased in the tissues of the fat-1 mice. Endogenous production of ω-3 PUFAs also markedly attenuated cytokine-induced activation of nuclear factor kappa-light-chain-enhancer of activated B cells (NF-κB) and extracellular signal–related kinase 1/2 (ERK1/2) (24, 30).

Our recent study and many other studies revealed that incubation of EPA and DHA with isolated human T cells could inhibit the proliferation of human T cells and their production of IL-2 (79–83). Similar findings were also made in the T-cells isolated from mice (80). *In vivo*, a dietary gain of ω-3 PUFAs has been shown to correct the imbalance of Th1 and Th2 ratios in such Th1-mediated autoimmune disease models as RA and EAE (84–86). Thus, both the proliferation and differentiation of T cells could be regulated by ω-3 PUFAs. EPA and DHA may have also influenced the function of antigen presenting cells. Studies derived from cultured cells have shown that the expression of major histocompatibility complex (MHC) II and antigen presentation via MHC II could be significantly reduced following the exposure to EPA and DHA (87–89). These findings are also supported by the observations in the mice fed fish-oil (90) or in humans with EPA/DHA supplementation (91). Taken together, these investigations supported the concept that ω-3 PUFAs can decrease levels of inflammatory cytokines, modulate T-cell differentiation, reduce antigen presentation via MHC II, and importantly correct a range of pathological conditions of RA.

## ω-3 PUFAs and Systemic Lupus Erythematosus

In addition to the investigations of ω-3 PUFAs in the treatment for RA, there have also been a number of studies in animal models and clinical trials assessing the effects of dietary supplementation of ω-3 PUFAs in other autoimmune diseases, such as SLE (42, 92). SLE is a common autoimmune disorder with diverse clinical manifestations including inflammation, blood vessel abnormalities, and immune-complex deposition, which are all associated with autoantibodies against cellular components (93, 94). Since the earliest clinical trial in 1989 (Table 2), there have been seven major published clinical studies focusing on the relationship between ω-3 PUFAs and SLE (42–48) (Table 2). All but one of the clinical studies (48) (Table 2) reported beneficial effects, including the improvement in endothelial function, disease activity, or inflammatory markers following the implementation of ω-3 PUFAs in SLE patients. In all of the studies with positive outcomes, more than 12 weeks of intervention appeared to be necessary. In a recent randomized placebo-controlled 6-month trial involving fifty SLE patients (47) (Table 2), the patients who received ω-3 PUFAs intervention (2.25 g EPA and 2.25 g DHA) showed remarkable improvement in their scores derived from Physician Global Assessment (PGA) and RAND 36-Item Short Form Health Survey (RAND SF-36, Version 1.0). The circulating levels of several inflammatory markers, including IL-13 and IL-12, were significantly decreased (47) (Table 2). A clinical nutritional study of SLE patients found that dietary patterns low in ω-3 PUFAs and high in carbohydrates positively correlated with the severity of disease activity, adverse serum lipids, and the presence of plaque (46) (Table 2). A double-blind, double placebo-controlled factorial trial in 52 patients with SLE (45) (Table 2) reported a significant decline in SLAM-R score (revised Systemic Lupus Activity Measure) from 6.12 to 4.69 in the subjects receiving EPA/DHA compared to those on placebo. In the study carried out by Das and colleagues (44) (Table 2), daily oral supplementation of even moderate EPA and DHA (EPA 162 mg, DHA 144 mg) induced prolonged remission of SLE in ten patients. Furthermore, EPA and DHA also suppressed both T-cell proliferation and the production of IL-1, IL-2, as well as TNF-α *in vitro* and *in vivo* (44) (Table 2). A comparative clinical study compared fatty acid (FA) compositions in the red blood cells (RBC) and plasma between the female SLE patients and age-matched females with a history of cardiovascular disease (CVD) or those SLE patients with no history of CVD (SLE+CVD and SLE-CVD) (5). The plasma levels of EPA and ω-3 index (EPA+DHA) were significantly lower in the SLE patients than the CVD controls. In RBC, the ratio of AA to EPA was also significantly higher in the SLE patients than in the controls. Thus, although there was no intervention with ω-3 PUFA supplement, these SLE patients clearly had altered plasma and RBC FA compositions favoring inflammation pathology (5). Following this study, Luca Navarini et al. analyzed fatty acids or metabolites in the plasma samples from SLE patients (95). RvD1, the main metabolic product of DHA, was found to be remarkably lower in the samples from SLE patients than those from the non-SLE controls (95), further supporting the potential of using ω-3 PUFAs and its metabolites in the clinical management of SLE.

**Table 2:** Interventional studies with ω-3 PUFAs in patients with systemic lupus erythematosus (SLE).

**Study**	***n***	**Study design**	**Results**
Clark et al. (42)	12 patients with SLE	12 patients took regular diet with two 1 g MaxEPA capsules (contained 180 mg EPA and 120 mg DHA) for 5 weeks, followed by a 5 weeks washout phase when no MaxEPA capsules were taken. Then the patients took six EPA capsules for 5 weeks.	Dietary supplementation with fish oil affects the mechanisms involved in inflammatory and atherosclerotic vascular disease in patients with lupus nephritis, including neutrophil leukotriene B4 release was reduced 78 and 42%, respectively, by the low and higher doses of fish oil. The higher fish oil dose induced a 38% decrease in triglyceride and a 39% reduction in VLDL cholesterol associated with a 28% rise in HDL, cholesterol. The fish oil had no effect on immune complex or anti-DNA antibody.
Westbergetal and Tarkowski (43)	17 patients with moderately active SLE	A double-blind, crossover study on the effect of MaxEPA (contained 180 mg EPA and 120 mg DHA), using olive oil as the control substance. 8/17 on MaxEPA, 2/17 on the control substance for 6 months.	MaxEPA had beneficial effects on the disease but short-lived.
Das et al. (44)	10 patients with newly diagnosed SLE	Patients were given orally 6 capsules of EPA/DHA (each contained EPA 27 mg, and DHA 24 mg) for 1 or 2 months. Some of these patients who had significant renal and/or other target organ involvement were given steroids initially for not more than l−2 months in addition to EPA/DHA. At the end of 1 or 2 months of therapy, steroids were withdrawn while patients continued to take EPA/DHA capsules.	Oral supplementation of EPA and DHA induced prolonged remission of SLE without any side-effects.
Duffy et al. (45)	52 patients with SLE	A double-blind, double placebo-controlled factorial trial for 24 weeks. One group received 3 g MaxEPA (contained 180 mg EPA and 120 mg DHA) and 3 mg copper, another 3 g MaxEPA and placebo copper, another 3 mg copper and placebo fish oil, and the fourth group received both placebo capsules.	Dietary supplementation with fish oil is beneficial in modifying symptomatic disease activity. There was a significant decline in SLAM-R score from 6.12 to 4.69 (*p* < 0.05) in those subjects taking fish oil compared to placebo. No significant effect on SLAM-R was observed in subjects taking copper.
Elkan et al. (46)	114 patients with SLE	In all 114 patients with SLE and 122 age- and sex-matched population-based controls answered a food frequency questionnaire (FFQ).	The low intake of ω-3 and high intake of carbohydrate among patients with SLE appear to be associated with worse disease activity, adverse serum lipids, and plaque presence.
Arriens et al. (47)	50 SLE patients	Fifty SLE patients were randomized 1:1 to fish oil supplementation or olive oil placebo, and blinded to their treatment group for 6 months.	Fish oil supplementation demonstrated improvement in PGA, RAND SF-36, and some circulating inflammatory markers of SLE patients.
Bello et al. (48)	85 SLE patients	SLE patients were randomly assigned to 3 g of ω-3 (Lovaza, GSK) vs. placebo for 12 weeks.	ω-3 did not improve endothelial function, disease activity, nor reduce inflammatory markers in SLE.

*SLE, Systemic lupus erythematosus; VLDL, Very low density lipoprotein; HDL, High density lipoprotein; PGA, Physician global assessment; RAND SF-36, RAND 36-Item short form health survey version 1.0; SLAM-R, Systemic lupus activity measure*.

Mounting evidence built in SLE animal models strongly supports the preventative and therapeutic benefits of ω-3 PUFAs against SLE. Although several SLE models have been widely used in published animal studies, we decided to focus on the results derived from NZB/NZW F1 mice (herein after named B/W F1) because it is a classic spontaneous SLE model with close resemblance to the development of human SLE. Fernandes et al. found that a diet rich in long chain ω-3 fatty acids (10%) delayed the onset, attenuated the severity of autoimmune diseases, and prolonged the survival of female B/W F1 mice (96). Such amelioration of autoimmune disease was mainly attributed to the elevation of transforming growth factor β1 (TGF-β1) and IL-4 as well as the reduction of pro-inflammatory cytokines IL-2 from the splenocytes of B/W F1 mice (96). Perhaps not surprisingly, in their following study, the analysis of fatty acids revealed significantly elevated EPA and DHA levels in the kidney tissue samples of the EPA/DHA-fed animals, and further confirmed the decreased expression of TGF-β1 played a key role in the development of SLE (97). In a separate independent study, the B/W F1 mice, after fed linseed-oil (containing 70% ω-3 PUFAs), showed lower titers of antibodies to DNA as well as cardiolipin and less severe kidney damage than the B/W F1 mice fed control diets (98). Consistent with these findings, Halade et al. found that the concentrated EPA/DHA (Lovaza®) had a dose-dependent therapeutic effect on SLE progression: the diet containing 1% Lovaza extending maximal lifespan to 517 days; and the diet containing 4% Lovaza further prolonged both the median (502 d) and maximal (600 d) life span in B/W F1 mice compared to the same mice fed standard chow diet (301 d) (99). In addition, the diet containing 4% Lovaza significantly decreased anti-dsDNA antibodies, glomerulonephritis, and pro-inflammatory cytokines (IL-1β, IL-6, TNF-α) in the splenocytes. Notably, NF-κB activation and p65 nuclear translocation were also lowered by 4% Lovaza-containing diet when compared to that in the control diet. An independent study by Pestka et al. found that ω-3 PUFAs-enriched diets delayed the onset and markedly attenuated the severity of autoimmune glomerulonephritis, plasma autoantibodies, and proteinuria in the B/W F1 mice (100); importantly, such suppression of autoimmunity was found to correlate with a generalized reduction of CD4^+^ T cell-specific gene expression in the kidneys and/or spleens of the SLE mice (100). Thus, daily oral supplementation of ω-3 PUFAs has shown promising therapeutic effects, even at low dosages, by suppressing glomerulonephritis and extending life span in patients and SLE-prone animals, most likely via reducing inflammatory cytokines. These studies further demonstrated that ω-3 PUFAs could alleviate the severity and slow the progression of SLE by regulating.

## ω-3 PUFAs and Type 1 Diabetes

T1D is a polygenic and organ-specific autoimmune disease, in which a certain subclass of T lymphocytes is involved in executing autoimmune attacks that lead to the destruction of pancreatic β cells (101, 102). Two recent large-scale clinical observational studies revealed clear preventative and therapeutic benefits against the development of T1D through early intervention with EPA/DHA-enriched agents to infants and children. Stene et al. initially reported in a large population-based case-control study that dietary supplement of cod-liver oil and vitamin D in the first year of life significantly reduced the incidence of childhood-onset T1D (49) (Table 3). In a separate longitudinal, observational study (part of the Diabetes Autoimmunity Study in the Young, DAISY), Norris and colleagues showed that EPA/DHA supplement starting from first year of life sharply lowered the incidence of islet autoimmunity and the titers of autoantibodies in children with high risk of T1D (50) (Table 3). In an attempt to treat a childhood onset T1D, Ricordi's group successfully normalized blood glucose and sharply reduced the dose of injected insulin to just once a day after a year and half of oral EPA/DHA and vitamin D supplement in an 8-year-old child (51) (Table 3). A multicenter, two-arm, randomized, double-blind pilot trial of DHA supplementation, beginning either in the last trimester of pregnancy (41 infants) or in the first 5 months after birth (57 infants) with a high genetic risk for T1D was conducted by Dr. Chase's group (52) (Table 3). There were no statistically significant reductions in the blood levels of inflammatory cytokines, such as IL-1β, TNF-α or interleukin-12 subunit p40 (IL-12p40). However, high-sensitivity C-reactive protein (hsCRP) was significantly lower in breast-fed DHA-treated infants compared to all formula-fed infants at age 12 months, suggesting that the nutritional matrix of breast milk, particularly the ones with increased DHA contents, has an anti-inflammatory effect (52) (Table 3).

**Table 3:** Interventional studies with ω-3 PUFAs in patients with type 1 diabetes (T1D).

**Study**	***n***	**Study design**	**Results**
Stene and Joner et al. (49)	545 subjects with childhood-onset T1D	Daily supplements with 5 mL of cod liver oil (0.6 g DHA, 0.4 g EPA, and 10 μg vitamin D) in the first year of life.	Significantly lower risk of type 1 diabetes in the first year of life with supplements of cod liver oil.
Norris et al. (50)	1770 children at increased risk for T1D	A longitudinal, observational study of children at increased risk for type1 diabetes with dietary intake of PUFAs (~150 mg/day EPA+DHA) started at age 1 yearThe mean age at follow-up was 6.2 years.	EPA/DHA supplement starting from the first year of life sharply lowered the incidence of islet autoimmunity and the titers of autoantibodies in children with a high risk of T1D.
Cadario et al. (51)	2 cases of T1D in pediatric subjects after a short clinical history of classic symptoms of overt disease	Supplements of vitamin D (1,000 IU/day) started just at the discharge and ω-3 (EPA + DHA 50–60 mg/kg/day, EnerZona^®^ω-3).	Improved blood glucose control and progressively reduced in relation to blood glucose eat awakening. A small amount of basal insulin at bedtime was maintained. Vitamin D and ω-3 supplementation may represent a cost-effective strategy in T1D.
Chase et al. (52)	Beginning either in the last trimester of pregnancy (41 infants) or in the first 5 months after birth (57 infants). Infants had a first-degree relative with T1D	Mothers received DHA (800 mg/day) or corn/soy oil (800 mg/day) in the last trimester of pregnancy and continued on this same dose after delivery if breast-feeding. Formula-fed infants received formula with 10.2 mg DHA/ounce (treatment) or 3.4 mg DHA/ounce (control).	The levels of RBC DHA increased in treated infants. No statistically significant reductions in the production of the inflammatory cytokines. Reduced hsCRP level in breast-fed DHA-treated infants.

*PUFAs, Polyunsaturated fatty acids; T1D, Type1 diabetes; DHA, Docosahexaenoic acid; EPA, Eicosapentaenoic acid; hsCRP, high-sensitivity C -reactive protein*.

A series of recent genetic and clinical studies have shed new light on the mechanisms and the therapeutic potential of ω-3 PUFAs in suppressing autoimmunity as well as protecting and enhancing islet functions. In animal studies, Otani et al. reported that low ratio of ω-6/ω-3 at 3.0 in the maternal diet during gestation and lactation delayed the onset of diabetes in NOD mice offspring (103). Their further study indicated that diet with a low ω-6/ω-3 ratio started immediately after the onset of overt diabetes prolonged survival of type 1 diabetes in NOD mice (104). However, a study by a different group failed to see a significant delay in the onset of diabetes with the supplementation of fish-oil (105). The author ascribed these results to the low power of statistical method and the possibility that EPA/DHA may have undergone oxidation after thawing from the −20°C storage condition (105). However, other factors, including the dosage and the concentrations of the administered EPA/DHA, should not be excluded.

The recent research coming from our own group revealed that elevation of cellular ω-3 PUFAs via the transgenic expression of fat-1 almost completely blocked the pancreatic β-cells from the attack of pro-inflammatory cytokines while significantly enhancing insulin secretion (30). These effects were primarily mediated via the reduction of PGE_2_ secretion from the islets and β-cells, suggesting that the control of inflammation through reduction of ω-6 PUFAs and their metabolites was the main theme in the regulation of β-cell functions (30). These findings derived from the fat-1 transgenic model was also consistent with the results from earlier nutritional studies that dietary gain of marine ω-3 PUFAs could restore palmitate acid-or LA-impaired insulin secretion (106, 107).

In our latest published report using the spontaneous type 1 diabetic NOD mice, an EPA/DHA-enriched diet and a fat-1 gene therapy not only delayed the onset but also reversed the autoimmunity and diabetes (80). Both methods of treatment, provided as either a preventative or a therapeutic modality, significantly suppressed Th1 and Th17 cell populations, reduced the secreted levels of IFN-γ and IL-17, increased the proportion of Th2 and regulatory T cells (Tregs). Thus, EPA/DHA-initiated global changes in CD4^+^ T-cells are likely the primary cellular mechanisms stalling the development of autoimmunity in T1D (80). Multiple molecular mechanisms may have contributed to the effects of ω-3 PUFAs on CD4^+^ T-cell differentiation. In particular, the metabolites of PUFAs played critical roles in the modulation of immune system. For example, arachidonic acid (an ω-6 PUFA)-derived eicosanoids, synthesized through the activities of such enzymes as lipoxygenase, cyclooxygenase, and cytochrome P450, often have a pro-inflammatory effect (108). In contrast, EPA/DHA-derived eicosanoids and docosanoids are less inflammatory than those from ω-6 PUFAs (16). In our analysis, different eicosanoids and docosanoids played distinct roles in CD4^+^ T-cell differentiation (80). Prostaglandin D3 (PGD3) (EPA-derived metabolite) had a strong inhibitory effect on Th1 and Th17 differentiation and increased the percentage of Th2 and Treg.17, 18-dihydroxy-5Z,8Z,11Z,14Z-eicosatetraenoic acid (17,18-DiHETE), another EPA-derived metabolite, only decreased the population of Th17. One of the DHA-derived metabolites, resolvin D1 (RvD1), had a strong inhibitory effect on Th1 differentiation and promoted Th2 and Tregs differentiation. In contrast, the pro-inflammatory eicosanoids (synthesized from AA), such as 15-Hydroxyeicosatetraenoic acid (15-HETE) and 20-Hydroxyeicosatetraenoic acid (20-HETE), had exactly the opposite effects on naive CD4^+^ T-cell differentiation than those of EPA/DHA-derived metabolites (80).

**Figure 1:**
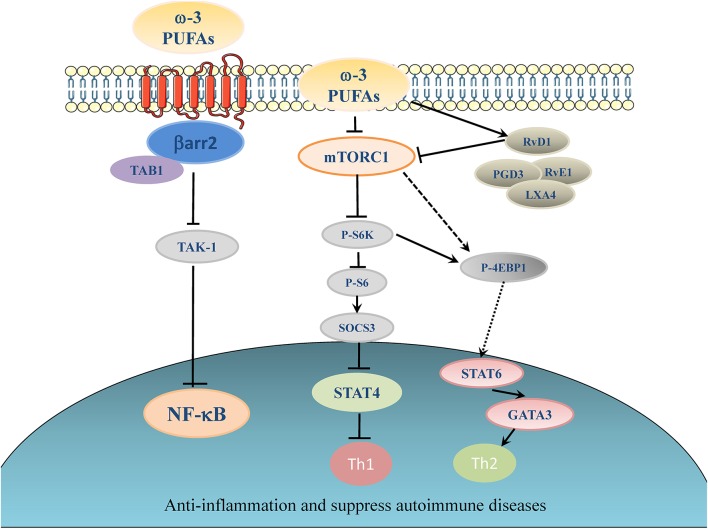
Proposed working model of ω-3 PUFAs induced inhibitory effect on inflammation and autoimmunity. Elevation of ω-3 PUFAs can stimulate one of its major receptors, GPR120. GPR120 couples to β-arrestin2 and inhibit TAB1-mediated activation of TAK1, providing a mechanism for inhibition of NF-κB related pro-inflammatory signaling pathways in autoimmune disease (103, 109). ω-3 PUFAs and their metabolites suppress the activation of Th1 cells through inhibiting of mTORC1 (80, 110, 111), which in turn activates suppressor of cytokine signaling (SOCS) (112). ω-3 PUFAs can inhibit signal transducer and activator of transcription (STAT) 4 which is essential for the development of Th1 cells from naive CD4^+^ T cells (113). In another signaling pathway, ω-3 PUFAs can dictate the differentiation toward Th2 cells by activating 4E-BP1 through dephosphorylation and then stimulate STAT6 (80).

Olefsky' group has identified a receptor for ω-3 PUFA, which is G-protein coupled receptor 120 (GPR120), that can mediate at least in part the anti-inflammation effect of ω-3 PUFAs (103, 109). GPR120 couples to β-arrestin2 and inhibits TAK1 binding protein 1 (TAB1)-mediated activation of transforming growth factor-β activated kinase 1 (TAK1), providing a mechanism for inhibition of both the NF-κB related Toll-like receptor (TLR) signaling and TNF-α pro-inflammatory signaling (103, 109) (see the proposed model in Figure 1). Deficiency of GPR120 in mice and humans produced symptoms very similar to those of wild-type mice fed LOW ω-3 PUFA diets, including reduced energy expenditure, obesity, insulin resistance, and an elevated state of inflammation (114).

Our recent studies further revealed that the mTOR has emerged as an important signaling switch for ω-3 PUFA-regulated differentiation of CD4^+^ T helper cells (110). In particular, application of rapamycin has been shown to inhibit the proliferation of T-cells and the differentiation to Th1 even in the presence of TCR co-stimulatory molecules, such as CD28 (82). T-cell specific mutation of mTOR gene or rapamycin treatment could suppress differentiation to Th17 cell (115). In contrast, rapamycin or diminution of mTOR activity promoted naive CD4^+^ T cells differentiating into Tregs (115). EPA/DHA and ω-6 PUFAs (particularly AA) had the opposite effects on mTORC1 signaling pathway. EPA/DHA reduced phosphorylation of ribosomal protein S6, indicative of inhibition of mTORC1 activity, whereas AA increased S6 protein phosphorylation levels by activating mTORC1. Moreover, rapamycin, the inhibitor of mTOR, completely blocked the AA-induced Th1 differentiation. Thus, the totally opposite impact of ω-6- and ω-3 PUFAs on the regulation of mTOR complexes serves a molecular switch to dictate the differentiation fate of CD4^+^ T cells following the treatment of PUFAs (80) (for a proposed working model, see Figure 1).

Interestingly, even in a chemically (*Streptozotocin*, STZ) induced diabetic model, the pancreatic enrichment of ω-3 PUFAs as a result of fat-1 expression could also prevent the development of diabetes (116). This dramatic effect was accompanied by the reduction of the expression of proinflammatory cytokine genes, the blockade of NF-κB activation, and a sharp suppression of proinflammatory PUFA-derived lipid mediators in the pancreas of the fat-1 mice (116). In contrast, the EPA-derived anti-inflammatory metabolites, lipoxin A4 (LXA4), and 18-hydroxy-5Z,8Z,11Z,14Z,16E-eicosapentaenoic acid (18-HEPE), were highly elevated in the STZ-treated fat-1 mice (116). Taken together, these clinical and animal studies have provided the hope that intervention with ω-3 PUFAs can be successfully applied to the prevention, and even therapeutic treatment of T1D. Additional studies are required to validate the effect of administration of ω-3 fatty acids on the progression of T1D.

Taken together, the reduction of ω-6 PUFAs and elevation of ω-3 PUFA intake are critical to the prevention and treatment of type 1 diabetes, which should help direct the future clinical intervention with EPA/DHA supplementation.

## ω-3 PUFAs and Multiple Sclerosis

MS is a chronic autoimmune inflammatory disease of the brain and spinal cord in which immune cell infiltration promotes inflammation, demyelination, and neuroaxonal degeneration, leading to the damage of neuronal signaling (117, 118). At the initial stages of the disease, most patients experience reversible neurological dysfunction, which typically lasts several days or weeks. Over time, irreversible clinical, and cognitive deficits develop (119, 120).

ω-3 PUFAs have attracted a great deal of attention as the potential agents in the clinical management of MS owing to their anti-inflammatory, antithrombotic, antioxidant, and immunomodulatory functions (118, 121–123). Indeed, largely inspired by Swank's observation that a higher incidence of MS occurred in Norwegians with diets of higher levels of animal fats and dairy products than those with diets of higher amounts of fish, several clinical studies have been conducted with the focus on dietary supplements like vitamin D, polyunsaturated fatty acids or other specific diets (124). A Norwegian intervention study of the patients with newly diagnosed MS reported that a 2-year treatment regimen with 0.9 g/day supplement of long chain marine fatty acids and vitamins showed a significant reduction in the mean annual exacerbation rate and the mean expanded disability status scale (EDSS) as compared to the pre-study values (53) (Table 4). In a randomized, placebo-controlled clinical trial with EDSS score and inflammation as the primary outcomes, a significant improvement in EDSS compared with placebo was observed in the patients taking ω-3 fatty acid plus vitamin D supplements (54) (Table 4). In a multi-center incident case-control study (involving 267 cases and 517 controls) conducted in four regions of Australia, high intake of ω-3 PUFAs was strongly associated with a decreased risk of a first clinical diagnosis of central nervous system (CNS) demyelination (55) (Table 4). In a double-blind and randomized trial for the duration of 1 year, 27 relapsing-remitting MS (RRMS) patients were treated with the same doses of ω-3 PUFAs and olive oil (56) (Table 4). Patients were randomly divided into two groups: the ω-3 group with dietary fat intake restricted to <15% of the daily calorie intake and the olive oil group with dietary fat intake to <30%. The results showed no significant effect on relapse rate or clinical severity (EDSS) scores following the 12-month intervention. However, the patients with relapsing-remitting MS benefited from a low fat diet supplemented with ω-3 PUFAs as the mean change of relapse rate in the ω-3 group: −0.797 relapses/year (*P* = 0.021) vs. −0.69 (*P* = 0.044) in the olive oil control group (56) (Table 4). However, not all clinical studies yielded positive outcomes. Torkildsen et al. evaluated the effect of ω-3 PUFAs on MS disease severity in a randomized, double-blind, and placebo-controlled trial (57) (Table 4). They applied 1,350 mg of EPA plus 850 mg of DHA daily in the treatment group with placebo as the control group for 6 months. Magnetic resonance imaging (MRI) was applied to quantify the number of new T1-weighted gadolinium-enhancing lesions during the first 6 months. Relapse rate, disability progression, fatigue, and quality of life were measured after 9 and 24 months. There was no difference between the ω-3 PUFAs and placebo groups during the first 6 months in the number of new gadolinium-enhancing MRI lesions, as well as in disease activity after 6 months or 24 months (57) (Table 4). It is still unclear about the discrepancy of outcomes in different trials although the dosage, the source and concentrations of EPA/DHA agents, the duration of the trial, and the methods of evaluation all could have led to the different results at the end of the trial.

**Table 4:** Interventional studies with ω-3 PUFAs in patients with multiple sclerosis (MS).

**Study**	***n***	**Study design**	**Results**
Nordvik et al. (53)	16 newly diagnosed MS patients with EDSS <6	Daily supplement with 5 ml fish oil (0.4 g EPA, 0.5 g DHA. 1.0 mg of vitamin A, 10 μg of vitamin D and 5.5 mg vitamin E). A vitamin B-complex (containing 2.25 mg of thiamin, 2.6 mg of riboflavin, 30 mg of niacin, 7 mg of pantothenic acid, 3 mg of pyridoxine, 150 μg of biotin, 100 μg of folic acid, 6 μg of cobalamin) and 200 mg of vitamin C (acid neutral).	ω-3 PUFAs supplementation given together with vitamins and dietary advice can improve clinical outcome in patients with newly diagnosed MS.
Kouchaki et al. (54)	53 patients with MS aged 18–55 y	Patients, aged 18–55 y, were matched for disease EDSS scores, gender, medications, BMI, and age (*n* = 53) and randomly received a combined 2 × 1,000 mg/d ω-3 fatty acid and 50,000 IU/biweekly cholecalciferol supplement or placebo for 12 week. The placebos were matched in color, shape, size, packaging, smell, and taste with supplements.	significant improvement in EDSS (β −0.18; 95% CI: −0.33, −0.04; *P* = 0.01) in patients taking ω-3 fatty acid plus vitamin D supplements compared with placebo.
Hoare et al. (55)	267 cases with FCD (aged 18–59) and 517 controls	Habitual dietary intake over the 12-month period prior to the study interview was collected from self-completion of the Cancer Council Victoria (CCV) Food Frequency Questionnaire (FFQ).	High intake of ω-3 PUFA and particularly that derived from fish rather than from plants was associated with a decreased risk of FCD.
Weinstock-Guttman et al. (56)	31 RRMS patients	Patients were randomized to two dietary interventions: the “Fish Oil” (FO) group received a low-fat diet (15% fat) with ω-3 FOs and the “Olive Oil” (OO) group received the AHA Step I diet (fat ≤ 30%) with OO supplements. The primary outcome measure was the Physical Components Scale (PCS) of the Short Health Status Questionnaire (SF-36).	Clinical benefits favoring the FO group were observed on PCS/SF-36 (*P* = 0.050) and MHI (*P* = 0.050) at 6 months. Reduced fatigue was seen on the OO diet at 6 months (*P* = 0.035). The relapse rate decreased in both groups relative to the rates during the 1 year preceding the study: mean change in relapse rate in the FO group: −0.79 ± SD 1.12 relapses/year (*P* = 0.021) vs. −0.69 ± SD 1.11 (*P* = 0.044) in the OO group.
Torkildsen et al. (57)	92 patients aged 18–55 years with active relapsing-remitting multiple sclerosis, with a disability score equivalent to 5.0 or less on the Kurtzke EDSS	92 patients were randomized to ω-3 fatty acids (*n* = 46) or placebo capsules (*n* = 46). Administration of 1,350 mg of eicosapentaenoic acid and 850 mg of docosahexaenoic acid daily or placebo. After 6 months, all patients also received subcutaneously 44 μg of IFNβ-1α 3 times per week for another 18 months.	No differences were detected in fatigue or quality-of-life scores, and no safety concerns appeared.

*PUFAs, Polyunsaturated fatty acids; DHA, Docosahexaenoic acid; EPA, Eicosapentaenoic acid; CNS, Central nervous system, FCD, a first clinical diagnosis of CNS demyelination; MS, Multiple sclerosis; RRMS, Relapsing/remitting multiple sclerosis; ATRA, All-trans retinoic acid; AOR, Adjusted odds ratio; CI, Confidence interval; EDSS, Expanded disability status scale; PCS, Physical components summary scale; SF-36, Short health status questionnaire; MHI, Mental health inventory; AHA, American heart association; IFNβ-1α, interferon beta– 1 alpha*.

Some of the positive clinical observations mentioned above prompted a series of cellular and animal research studies about the effects of ω-3 PUFAs on MS development. As described above, several different groups (including our own) have reported that ω-3 PUFAs could inhibit the activation of NF-κB via increasing the expression of PPARs (24, 30, 125–128), which in turn reduces the production of inflammatory markers. In a combinatorial treatment with DHA and all-trans-retinoic acid (a bioactive derivative of vitamin A), the expression of IL-17 and RORγt gene, which is vital for the initiation and progression of MS, was significantly attenuated in the PBMCs isolated from the patients with relapsing-remitting multiple sclerosis (129). Addition of EPA and DHA in cultured cells significantly decreased the production and activity of matrix metalloproteinase-9 (MMP-9) and inhibited the migration of human T cell derived from the PBMCs from MS patients, which implied the reduction of infiltrating capability of T-cells (130).

Kong et al. first reported in 2011 that dietary ω-3 fatty acids could suppress EAE in an animal model (73). Their extensive work showed that pretreatment of bone marrow-derived dendritic cells (DC) with DHA could prevent LPS-induced DC maturation and weaken the stimulation of antigen-specific T cells (73). Consistent with these *in vitro* observations, the results from the animal model further demonstrated decreased numbers of IFNγ- and IL-17-producing CD4^+^ T cells in both spleen and CNS (73). In another independent study, the mice with myelin oligodendrocyte glycoprotein (MOG)-induced EAE showed a significant delay in onset and amelioration in severity by DHA-enriched diet (131). In such context, the female mice were fed 4 weeks of either the control diet, or the diet containing 0.3 or 1.0% DHA in either phospholipid (PL) form or triacylglycerol (TAG) form pre-EAE induction and for 4 weeks post-EAE induction. The TAG-form DHA appeared to be effective in lower daily scores in the early phase of EAE during day 9–16 while PL-form DHA was effective in decreasing daily scores in the final outcome during day 23–28 (131). Increasing EPA content in the diet appeared to have the benefit similar to that of DHA-enriched diet. Unoda et al. found that EPA-enriched diet significantly lowered clinical EAE scores, and remarkably inhibited the production of IFN-γ and IL-17 (132). Consistent with these findings in the nutritional intervention studies, modeling of MS with cuprizone in the fat-1 mice revealed that endogenous production of ω-3 PUFAs strongly promoted remyelination after a toxic injury to CNS oligodendrocytes via modulation of the immune system (133, 134). Interestingly, some of the protective effects were mediated at least in part through EPA-derived lipid metabolites, such as 18-HEPE by acting directly on oligodendrocytes and neurons (133, 134). Taken together, the findings coming out of the clinical investigations and animal models provided the hope of applying high dose of EPA/DHA agents in the treatment of MS.

## Conclusion

The promising findings coming from the cumulative research work over the last decade solidified the role of ω-3 PUFAs as a potential candidate to prevent or even treat such autoimmune diseases as type 1 diabetes, RA, SLE, MS. Undoubtedly, many of the beneficial effects of ω-3 PUFAs can be traced back to their anti-inflammation actions; however, other mechanisms, such as the regulation of mTOR activity may also be in place. Although still facing some technical issues, particularly clinical evaluation of efficacy and safety, the use of high dosages of EPA/DHA or fat-1 gene therapy to generate endogenousω-3 PUFAs have a huge potential in the clinical treatment of these debilitating diseases.

## Author Contributions

XB and XL wrote the manuscript. AZ oversaw the editing. XL, SW, ZZ, FL, and AZ revised the manuscript. All authors read and approved the final manuscript.

### Conflict of Interest

The authors declare that the research was conducted in the absence of any commercial or financial relationships that could be construed as a potential conflict of interest.
